# Structure‐Function Relationship of Highly Reactive CuO_x_ Clusters on Co_3_O_4_ for Selective Formaldehyde Sensing at Low Temperatures

**DOI:** 10.1002/advs.202308224

**Published:** 2023-12-24

**Authors:** Matteo D'Andria, Frank Krumeich, Zhangyi Yao, Feng Ryan Wang, Andreas T. Güntner

**Affiliations:** ^1^ Human‐centered Sensing Laboratory, Department of Mechanical and Process Engineering, ETH Zurich Zurich CH‐8092 Switzerland; ^2^ Department of Chemistry and Applied Biosciences Laboratory of Inorganic Chemistry, ETH Zurich Zurich CH‐8093 Switzerland; ^3^ Department of Chemical Engineering University College London London WC1E 7JE UK

**Keywords:** clusters, inorganic catalysis, molecular sensing, nanotechnology, semiconductors, surface engineering

## Abstract

Designing reactive surface clusters at the nanoscale on metal‐oxide supports enables selective molecular interactions in low‐temperature catalysis and chemical sensing. Yet, finding effective material combinations and identifying the reactive site remains challenging and an obstacle for rational catalyst/sensor design. Here, the low‐temperature oxidation of formaldehyde with CuO_x_ clusters on Co_3_O_4_ nanoparticles is demonstrated yielding an excellent sensor for this critical air pollutant. When fabricated by flame‐aerosol technology, such CuO_x_ clusters are finely dispersed, while some Cu ions are incorporated into the Co_3_O_4_ lattice enhancing thermal stability. Importantly, infrared spectroscopy of adsorbed CO, near edge X‐ray absorption fine structure spectroscopy and temperature‐programmed reduction in H_2_ identified Cu^+^ and Cu^2+^ species in these clusters as active sites. Remarkably, the Cu^+^ surface concentration correlated with the apparent activation energy of formaldehyde oxidation (Spearman's coefficient *ρ* = 0.89) and sensor response (0.96), rendering it a performance descriptor. At optimal composition, such sensors detected even the lowest formaldehyde levels of 3 parts‐per‐billion (ppb) at 75°C, superior to state‐of‐the‐art sensors. Also, selectivity to other aldehydes, ketones, alcohols, and inorganic compounds, robustness to humidity and stable performance over 4 weeks are achieved, rendering such sensors promising as gas detectors in health monitoring, air and food quality control.

## Graphical abstract

Fine CuO_x_ clusters are dispersed on Co_3_O_4_ nanoparticles revealing outstanding formaldehyde oxidation properties even at low temperatures (e.g., 75 °C). These enable selective and highly sensitive formaldehyde detection down to 3 ppb in the presence of humidity. Cu^+^ is identified as a performance descriptor, correlating well with sensor response and oxidation kinetics.

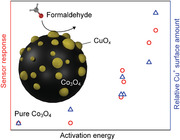

## 1 Introduction

Cluster design on metal oxides (MO_x_) is an emerging topic in heterogeneous catalysis^[^ ^1^ ^]^ and molecular sensing^[^ ^2^ ^]^ and has already led to exciting performance concerning low‐temperature reactivity^[^ ^3^ ^]^ and selectivity.^[^ ^1^, ^4^ ^]^ Enabled by advances in fabrication methods, it is possible today to populate clusters with finely tuned geometries, compositions and sizes down to isolated single atoms onto MO_x_ surfaces.^[^ ^5^ ^]^ Yet, finding suitable material combinations and understanding the complex electronic metal‐support interactions (EMSI)^[^ ^6^ ^]^ remains challenging but is required to tailor the active site speciation and find novel synergetic interactions with improved oxidation chemistry.^[^ ^7^, ^8^ ^]^

Chemoresistive sensing technology based on semiconductive MO_x_ nanoparticles benefits directly from such advances to address the pressing need for sensitive and highly selective molecular detectors in air quality assessment,^[^ ^9^ ^]^ food control^[^ ^10^ ^]^ and medical diagnostics,^[^ ^11^ ^]^ among other applications. To reduce the operational temperature for low‐power sensing, MO_x_ are often loaded with noble‐metal (NM) clusters (e.g., PdO_x_ on SnO_2_^[^ ^12^ ^]^). Enhanced sensing performance is associated with the combined result of electronic and chemical sensitization,^[^ ^13^ ^]^ i.e., the control over Fermi‐energy level^[^ ^14^, ^15^ ^]^ and spillover effect,^[^ ^16^ ^]^ respectively. Although NM‐loading typically yields improved sensitivity and lower operational temperatures,^[^ ^12^ ^]^ indiscriminate reduction of the energy barrier triggers the unspecific dissociation of various molecules on the sensor's surface compromising its selectivity.

Compared to usually applied Pd, Rh and other platinum‐group‐metals, non‐noble Cu is promising for selective oxidations owed to the lower position of its *d*‐band center.^[^ ^17^ ^]^ For instance, dispersed Cu‐species and clusters on the surface of CeO_2_ showed superior activity in the selective oxidation of CO.^[^ ^18^ ^]^ Also the supporting MO_x_ plays a major role,^[^ ^19^ ^]^ specifically its oxygen storage capacity^[^ ^20^ ^]^ and reducibility.^[^ ^21^ ^]^ Co_3_O_4_ is interesting due to the redox‐active Co^3+^/Co^2+^ pair,^[^ ^22^ ^]^ which enables the synthesis of O‐vacancy rich, non stoichiometric Co_3_O_4‐x_.^[^ ^21^ ^]^ This partial reduction of Co_3_O_4_ allows superior performance both as a catalyst to remove formaldehyde at room temperature,^[^ ^23^ ^]^ and as a sensor to detect it down to 50 ppb, at 225°C though.^[^ ^24^ ^]^

Here, we design and investigate flame‐aerosol‐made CuO_x_ clusters on Co_3_O_4_ and apply them for formaldehyde detection at low temperatures. Due to its carcinogenic nature, formaldehyde is of high relevance for air quality monitoring,^[^ ^25^ ^]^ and strict exposure limits apply for most countries (e.g., 8 ppb in France^[^ ^26^ ^]^). An overview over state‐of‐the‐art chemoresistive formaldehyde sensors is provided in **Table** **1**. These Cu/Co_3_O_4_ heterostructures are produced by flame spray pyrolysis (FSP), yielding materials with a high specific surface area. Detailed crystallographic analysis is performed to identify crystal phase dynamics and lattice‐incorporated Cu ions. By a combination of in situ infrared spectroscopy of adsorbed CO, near ambient pressure‐near edge X‐ray absorption fine structure (NAP‐NEXAFS) spectroscopy, H_2_‐temperature‐programmed reduction and oxidation kinetic analysis, the reactive site is investigated to reveal structure‐function relationships. Self‐assembling these nanoparticles into chemoresistive films enables their evaluation as formaldehyde sensors at ppb concentration with relative humidity and various confounders to meet the regulations in air quality monitoring. Finally, the best‐performing sensor is benchmarked to the state‐of‐the‐art.

**Table 1:** Chemoresistive formaldehyde sensors that work below 120°C in the presence of humidity.

Chemo – resistive material	LOQ^a)^ (RH), ppb	RH range	Formaldehyde selectivity to:						Operating temperature, [°C]	Ref.
			Acetone	Toluene	Ethanol	NH_3_	CO	CH_4_		
Ag/In_2_O_3_	50 (16%)	16‐75%	> 100	70	4	40	–	–	30	[99]
Ni‐doped In_2_O_3_/WS_2_	50 (dry)	0‐97%	3.3	–	2	1.7	–	4	25	[69]
Au@SnO_2_ core‐shell structures	20 000 (50%)	40‐70%	–	–	2.6	–	–	–	25	[85]
3DOM^b)^ Au/SnO_2_	10 (dry)	32‐91%	> 10	> 10	> 10	–	–	–	110	[70]
PdAu / SnO_2_ nanosheets	1 000 (40%)	40‐70%	8.9	9.1	5.2	9	–	–	110	[100]
NiO‐SnO_2_ nano – spheres	500 (dry)	0‐100%	10	–	5	–	–	–	100	[101]
GO/SnO_2_ hollow nanofibers	500 (31%)	31‐38%	12	30	2.6	30	–	–	120	[102]
CuO_x_ clusters / Co_3_O_4_	3 (50%)	0‐90%	7.3	19	5.5	58	89	52	75	This work

^a)^ Limit of quantification, i.e., lower quantified concentration;

^b)^ 3D ordered macroporous (3DOM).

## 2 Results and Discussion

### 2.1 CuO_x_ / Co_3_O_4_ Heterostructure Design and Synthesis

**Figure 1:**
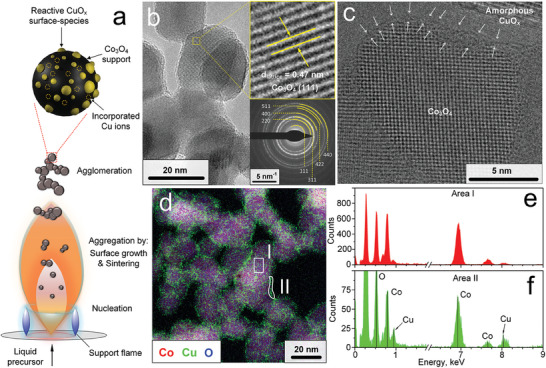
a) Schematic FSP reactor for Co_3_O_4_ nanoparticle synthesis with incorporated Cu ions and CuO_x_ surface clusters. b) TEM image of 5 wt.% Cu/Co_3_O_4_ nanoparticles after annealing at 500°C for 5 h with visible lattice fringes and measured lattice spacing (inset, top‐right). SAED pattern of the same powder with indicated Miller indices of cubic Co_3_O_4_ (inset, bottom‐right). c) HR‐TEM image of such nanoparticles with indicated amorphous CuO_x_ clusters. d) Elemental map of 5 wt.% Cu/Co_3_O_4_ with the distribution of Co (red), Cu (green) and O (blue). EDXS spectra of e) area I and f) II, as indicated in (d), respectively.

We utilize flame‐aerosol technology to form CuO_x_ clusters on Co_3_O_4_ nanoparticles, as previously done on CeO_2_.^[^ ^17^ ^]^ Thereby, a liquid organometallic precursor with homogeneously mixed Cu and Co ions is dispersed with oxygen, evaporated and combusted under atmospheric conditions. As sketched in **Figure** **1**a, nanoparticles are formed by gas‐solid conversion through nucleation before growing by coagulation and sintering to form agglomerated structures.^[^ ^27^ ^]^ At Co‐rich conditions, primarily Co_x_O_y_ particles should be formed, and Cu species may condense on their surfaces, forming clusters.^[^ ^28^ ^]^ Additionally, some Cu ions present in the flame may be incorporated into the Co_x_O_y_ lattice of particle nuclei, as explored below. Rapid quenching of the product aerosol preserves the nanostructured morphology of the obtained particles.^[^ ^29^ ^]^

Figure 1b shows a transmission electron microscopy (TEM) image of 5 wt.% Cu/Co_3_O_4_ nanoparticles after annealing them at 500°C for 5 h. Particles with high crystallinity are visible, as supported by their faceted shape with clearly visible lattice fringes (inset top‐right). The measured lattice spacing of 0.47 nm matches the (111) crystal plane of cubic Co_3_O_4_.^[^ ^30^ ^]^ This is further confirmed by the selected area electron diffraction (SAED) in Figure 1b (inset bottom‐right) with patterns associated with the (111), (220), (311), (400), (422), (511) and (440) planes, respectively. Such cubic Co_3_O_4_ is stable at room temperature^[^ ^31^ ^]^ and has been previously obtained by flame‐aerosol technology, applying different precursor formulations.^[^ ^32^ ^]^

The Co_3_O_4_ nanoparticles are populated with CuO_x_ clusters, as revealed by high resolution TEM (HR‐TEM) in Figure 1c. These clusters feature no visible lattice fringes, indicating their amorphous structure. Note that some Co_3_O_4_ surfaces are free of such clusters (Figure S1a, Supporting Information), suggesting an inhomogeneous distribution of the CuO_x_. No such clusters are observed for pure Co_3_O_4_ (Figure S1b, Supporting Information), where lattice fringes extend until the nanoparticle edges. No carbon contamination^[^ ^33^, ^34^ ^]^ is observed, that usually forms hermetic coatings around nanoparticles.

The presence of Cu is confirmed by elemental mapping (Figure 1d), which shows that Co and O are distributed over the Co_3_O_4_ particles (refer to Figure S2, Supporting Information for maps of individual elements). The Cu (green) seems to be enriched on the surface of the particles. This is confirmed by energy‐dispersive X‐ray spectroscopy (EDXS) of selected areas (Figure 1e,f) in Figure 1d, that indicate a higher Cu content (elevated peaks at 0.90 and 8.05 keV) at the rim of Co_3_O_4_ particles. Such CuO_x_ enrichment on surfaces was also observed for as‐prepared powders (i.e., before annealing, Figure S3, Supporting Information).

### 2.2 Crystal Structure and Cu Incorporation

**Figure 2:**
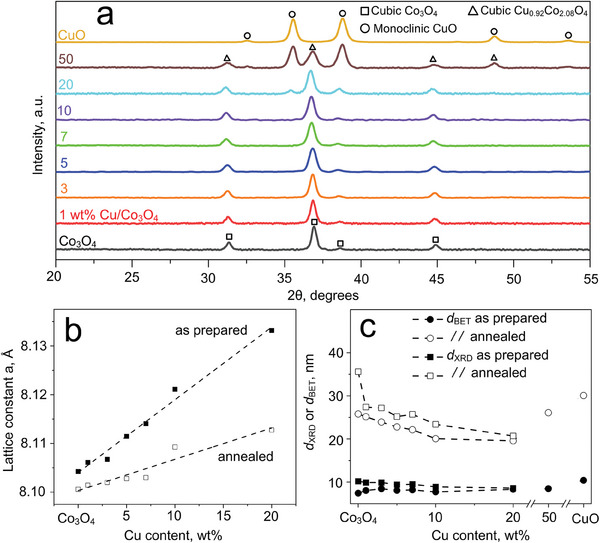
a) XRD patterns of pure Co_3_O_4_, CuO and Cu/Co_3_O_4_ powders after annealing for 5 h at 500°C in air. Indicated are the reference peaks for cubic Co_3_O_4_ (squares), monoclinic CuO (circles) and cubic Cu_0.92_Co_2.08_O_4_ (triangles), with powder diffraction files (PDF) being provided in the Experimental Section. b) Lattice constant *a* of cubic Co_3_O_4_ as a function of the Cu content for as‐prepared (filled squares) and annealed (open squares) powders. The dashed lines indicate linear fits. c) Co_3_O_4_ crystal (*d*_XRD_, squares) and BET‐equivalent particle (*d*_BET_, circles) diameters as a function of Cu content for as prepared (filled symbols) and annealed (open symbols) powders. Note that Co_3_O_4_ crystal sizes for 50 wt.% Cu/Co_3_O_4_ and pure CuO were not determined due to multiple phases with overlapping XRD peaks or absence of that phase, respectively.

**Figure** **2**a shows the X‐ray diffraction (XRD) patterns of annealed pure Co_3_O_4_, CuO and Cu/Co_3_O_4_ powders. Note that the corresponding patterns for as‐prepared powders are reproduced in Figure S4 (Supporting Information). Co_3_O_4_ is highly crystalline and forms the cubic spinel phase (squares), in agreement with TEM and electron diffraction (Figure 1b). Most importantly, this crystal phase does not change for up to a nominal 10 wt.% Cu. The CuO_x_ clusters observed by elemental mapping (Figure 1d–f) are not visible, due to their crystal size below the XRD detection limit (i.e., 5 nm^[^ ^35^ ^]^) and an amorphous structure. Only at 20 wt.% Cu, first peaks associated with monoclinic CuO (circles) occur at 2θ = 35.6°, 38.8° that become more pronounced at 50 wt.%. Also some spinel Cu_0.92_Co_2.08_O_4_ (triangles) may be present,^[^ ^36^ ^]^ featuring overlapping peaks with CuO and Co_3_O_4_. In contrast, metastable orthorhombic Cu_2_CoO_3_ (Figure S4, Supporting Information) was observed for as‐prepared samples. This phase is thermodynamically stable only in the range of 900 – 1000°C^[^ ^37^ ^]^ and is formed during flame‐aerosol synthesis due to the high temperature and rapid quenching of product nanoparticles, as similarly observed for metastable *ε*‐WO_3_^[^ ^38^ ^]^ and BaCO_3_.^[^ ^39^ ^]^ Finally, the monoclinic phase is obtained for pure CuO, in agreement with literature.^[^ ^40^ ^]^

To investigate a possible Cu ion incorporation into Co_3_O_4_, alterations of the lattice parameters are investigated by analyzing shifts of the Co_3_O_4_ main peak at 2θ = 36.9° (see also Figure S5, Supporting Information), after correcting for sample displacement with an internal standard^[^ ^35^ ^]^ (i.e., SnTe). The lattice constant *a* for as‐prepared pure Co_3_O_4_ is 8.104 Å (Figure 2b) and thus somewhat larger than the crystallographic reference for Co_3_O_4_ single crystals (8.084 Å^[^ ^31^ ^]^). Such deviation can be explained by the flame aerosol synthesis, which results in nanoparticles with high defect concentration and residual lattice strains.^[^ ^41^ ^]^ Most importantly, when increasing the Cu content (filled squares) to 20 wt.%, the lattice constant *a* grows to 8.133 Å, following a linear increase in agreement with Vegard's rule.^[^ ^42^ ^]^ This observation indicates a lattice expansion and suggests the incorporation of Cu substituting Co ions in the Co_3_O_4_ lattice, with both cations featuring similar ionic radii.^[^ ^43^ ^]^ Similar incorporation has been observed during flame‐aerosol synthesis of other material compositions (e.g., Ti/ZnO,^[^ ^44^ ^]^ Y/ZnO,^[^ ^45^ ^]^ Si/MoO_3_^[^ ^46^ ^]^) and should be associated with the high mobility of metal ions during nucleation at the high flame temperatures followed by rapid quenching.^[^ ^47^ ^]^

Remarkably, the lattice parameter *a* decreases consistently for all Cu contents during annealing (empty symbols, Figure 2b) compared to as‐prepared samples (filled symbols). Specifically, for pure Co_3_O_4_, the lattice constant *a* is now 8.101 Å, which is closer to the reference value, as expected due to temperature‐induced lattice‐strain relaxations.^[^ ^48^ ^]^ When adding Cu, the lattice constant *a* still increases, suggesting that some substitutionally incorporated Cu ions are still present, but lower expansion hints at Cu migration to the surface, similarly to other Cu/Co_3_O_4_ materials after repeated heating cycles.^[^ ^49^ ^]^ As a result, annealing likely enriches the Co_3_O_4_ surface with Cu‐related species, which is favorable for surface‐active processes (e.g., gas sensing or heterogeneous catalysis), as explained below.

We also evaluated the crystal (*d*_XRD_, squares in Figure 2c) and BET‐equivalent particle (*d*_BET_, circles) sizes for as‐prepared (filled symbols) and annealed (empty symbols) powders. As‐prepared crystal and particle sizes of pure Co_3_O_4_, CuO, and Cu/Co_3_O_4_ are quite comparable, suggesting monocrystallinity, and they range from 7 to 11 nm. After annealing, the pure Co_3_O_4_ crystals grow to 36 nm, due to temperature‐activated crystal growth. The introduction of as little as nominal 1 wt.% Cu reduces the size to 27 nm, which further decreases to 21 nm when adding up to 20 wt.%. This further supports the substitutional incorporation of Cu ions forming defects that thermally stabilize the Co_3_O_4_ crystals, in agreement with literature.^[^ ^46^ ^]^ Note that the crystallite size of Co_3_O_4_ was not determined for 50 wt.% Cu due to the presence of multiple phases with overlapping diffraction peaks. As‐prepared monoclinic CuO features 10.7 nm large crystals, in agreement with the literature,^[^ ^40^ ^]^ that increase to 21.9 nm after annealing.

### 2.3 Surface Speciation and Redox Properties

**Figure 3:**
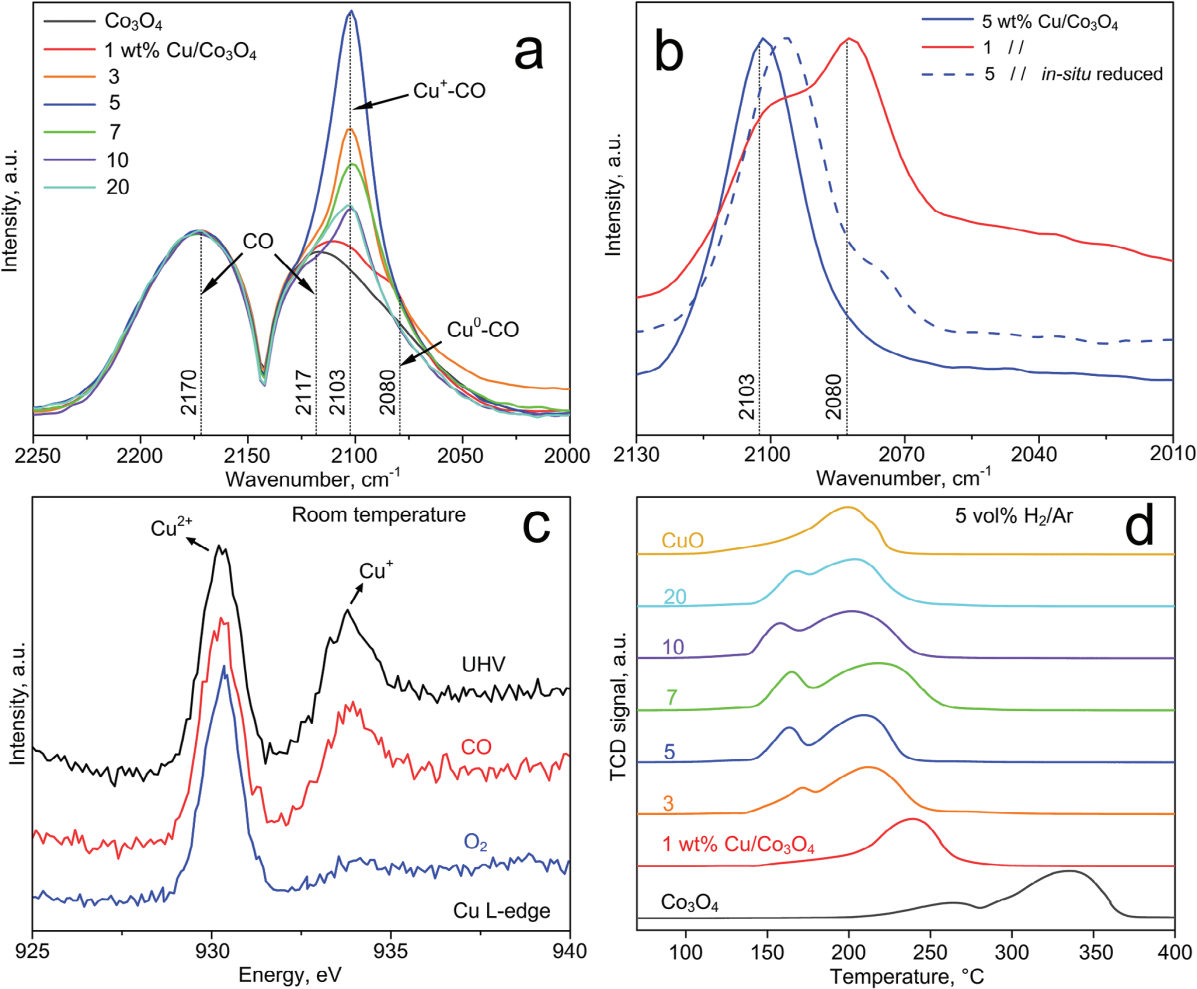
a) IR spectra of CO with 0 – 20 wt.% Cu/Co_3_O_4_ powders after annealing. Note that all spectra are normalized to the peak at 2170 cm^−1^ associated to gaseous CO. Indicated as vertical dashed lines are the normal stretches of gaseous CO at 2170 and 2117 cm^−1^, and the vibration of CO bonding with surface Cu^+^ and Cu^0^ at 2103 and 2080 cm^−1^, respectively. b) 1 wt.% (red) and 5 wt.% (blue, solid line) Cu/Co_3_O_4_ spectra after subtracting the gaseous interference as CO background (Figure S6b, Supporting Information), as well as the 5 wt.% powder after a 30 min in situ reduction with H_2_ at 375°C (blue, dashed line). c) NAP‐NEXAFS spectra of Cu L_3_‐edge (AEY mode) of 5 wt.% Cu/Co_3_O_4_ nanoparticles under UHV (black line), CO (red) and O_2_ (blue) at 298K. Indicated are the peaks associated with the presence of Cu^2+^ and Cu^+^ at approx. 930 and 934 eV, respectively. d) TPR profiles of such Cu/Co_3_O_4_ powders under 5 vol% H_2_/Ar between 75 – 400°C.

**Figure** **3**a shows the IR spectra of annealed 0 – 20 wt.% Cu/Co_3_O_4_ powders after exposure to CO, as obtained by in situ diffuse reflectance infrared Fourier transform spectroscopy (DRIFTS). A CO exposure time of 30 min has been chosen as it is sufficiently long to obtain saturation (Figure S6a, Supporting Information). Pure Co_3_O_4_ (black line) features two bands with peaks at 2170 and 2117 cm^−1^ that are associated^[^ ^50^ ^]^ with gaseous CO (Figure S6b, Supporting Information). Note that all spectra were aligned and normalized to the peak of the 2170 cm^−1^ vibration mode to allow a comparison of relative amounts of surface sites.^[^ ^51^ ^]^ Importantly, a new stretch at 2103 cm^−1^ emerges with increasing Cu content, which has been assigned to Cu^+^‐CO complex vibration.^[^ ^52^ ^]^ The CO may be bonded to atomic Cu sites,^[^ ^53^ ^]^ or to Cu clusters/nanoparticles.^[^ ^54^ ^]^ A maximum is reached at 5 wt.% Cu (blue curve), indicating the highest surface concentration of Cu^+^ active sites available for CO adsorption. At higher Cu content, this peak decreases, which might be related to the presence of larger CuO_x_ clusters with less surface area and/or a lower ratio of oxidized‐to‐metallic Cu inside the cluster. The latter parameter can compromise the activity of heterogeneous catalysts.^[^ ^55^ ^]^

Noteworthy, an additional shoulder with a peak at 2080 cm^−1^ is observed at a nominal 1 wt.% Cu. This is better visible in Figure 3b (solid red line), in which the spectrum of gaseous CO as background has been removed. This peak has been assigned to Cu^0^‐CO complex vibration,^[^ ^56^ ^]^ suggesting the presence of metallic Cu on the surface. A similar feature, though at a lower intensity, also appears for 5 wt.% Cu/Co_3_O_4_ after 30 min reduction in H_2_ at 375°C (dashed blue line) while it is hardly visible without reduction (solid blue line), suggesting the reducibility of Cu^+^ to Cu^0^. It is worth noting that the adsorption energy of CO on metallic Cu^0^ is lower than that on oxidized Cu^+^ sites.^[^ ^57^ ^]^ Thereby, Cu^0^ might also be also in 3 – 20 wt.% Cu/Co_3_O_4_ powders in small amounts, likely forming nanoparticles instead of single‐atom sites, as observed for Cu on CeO_2_.^[^ ^58^ ^]^ Furthermore, also Cu^2+^ might be present, that usually comes together with Cu^+^ in such clusters,^[^ ^59^ ^]^ but it is not observed by in situ DRIFTS under CO exposure due to the even lower adsorption energy of Cu^2+^‐CO.^[^ ^60^ ^]^

To explore the valence of Cu (i.e., oxidation states) in the CuO_x_ clusters further, we performed NAP‐NEXAFS spectroscopy of 5 wt.% Cu/Co_3_O_4_ nanoparticles at room temperature. At the Cu L‐edge, Cu^2+^ and Cu^+^ sites are identified^[^ ^59^ ^]^ by peaks at around 930 eV (2p to 3d transition) and 934 eV, respectively. Under ultra‐high vacuum (UHV, Figure 3c, black line) and CO (red line), both Cu^+^ and Cu^2+^ are present, in line with previous findings for CuO_x_ clusters on TiO_2_.^[^ ^61^ ^]^ When changing the atmosphere to O_2_, Cu^2+^ dominates indicating the oxidation Cu^+^ → Cu^2+^. These results suggest the co‐presence of Cu^+^ and Cu^2+^ in the CuO_x_ clusters and their compositional dependence on the surrounding atmosphere.

To investigate the reducibility of our Cu/Co_3_O_4_ samples further, we performed temperature‐programmed reduction using H_2_ (H_2_‐TPR). As shown in Figure 3d, pure Co_3_O_4_ features two well‐defined bands at approx. 260 and 330°C. These correspond to the Co^3+^ → Co^2+^ and Co^2+^ → Co^0^ sequential reductions.^[^ ^23^ ^]^ Pure CuO is reduced with peaks at 170 and 215°C, marking the sequential reductions Cu^2+^ → Cu^+^ and Cu^+^ → Cu^0^, respectively.^[^ ^62^ ^]^ Most importantly, the addition of Cu significantly reduces the reduction temperature of Co_3_O_4_. In fact, a new peak forms at 160°C with the highest intensity at nominal 5 – 10 wt.% Cu, indicating excellent H_2_‐reducibility at such compositions. Such enhanced low‐temperature reducibility should be associated with the presence of Cu^+^ and Cu^2+^ in these surface clusters (Figure 1d and Figure 3a–c), as similarly reported for CuO_x_ on TiO_2_.^[^ ^20^ ^]^ The broad TPR band between 170 – 240°C suggests the reduction of Cu – [O_x_] – Co^α+^ substituted solid solutions (as similarly reported for, e.g., Cu/CeO_2_^[^ ^17^, ^63^, ^64^ ^]^ and Cu/TiO_2_^[^ ^20^ ^]^) with α = 2, 3, and deconvoluted TPR profiles are shown in Figure S7 (Supporting Information).

### 2.4 Catalyst and Sensor Evaluation: Structure‐Function Relationship

**Figure 4:**
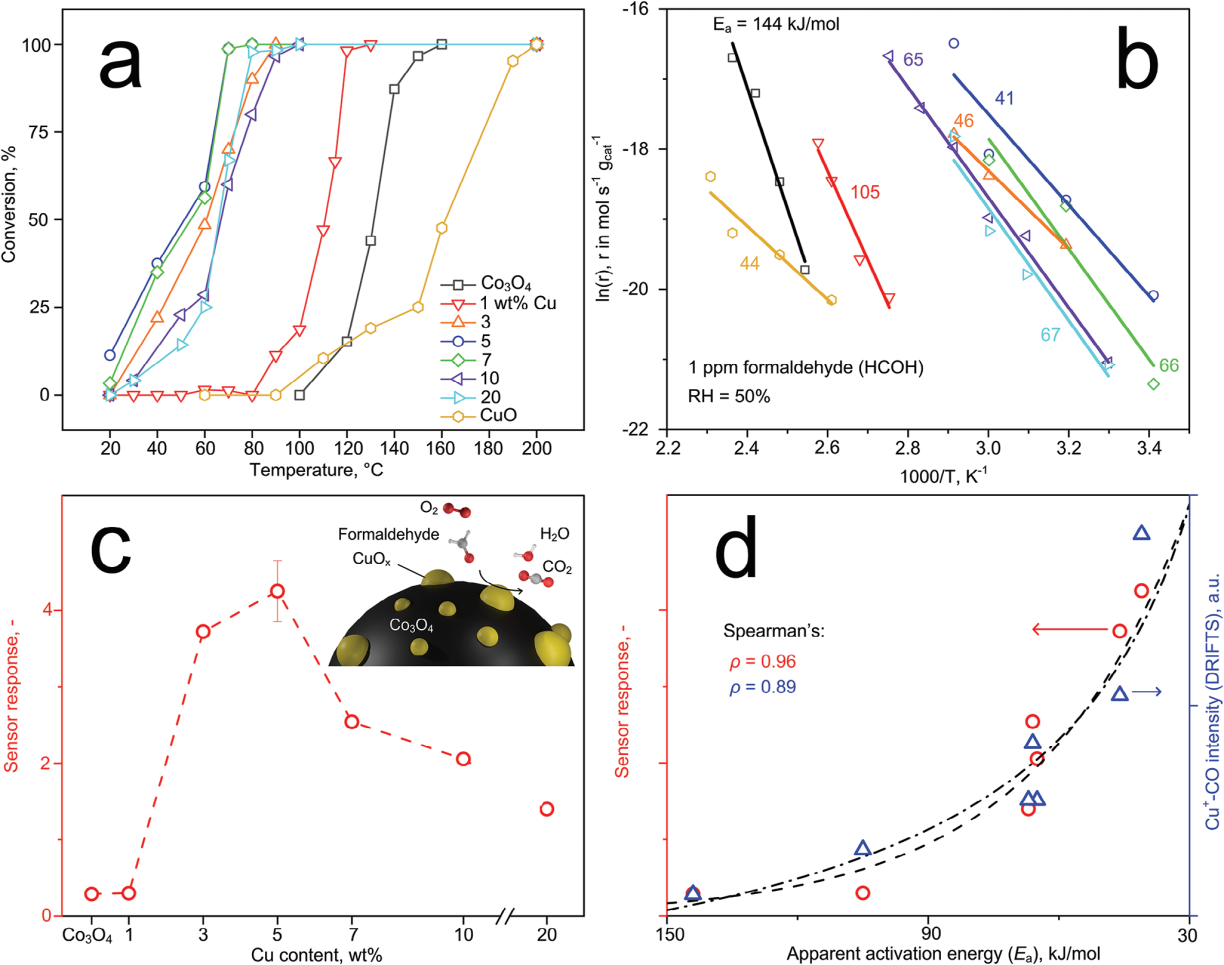
a) Catalytic conversion of 1 ppm formaldehyde over pure Co_3_O_4_, CuO and Cu/Co_3_O_4_ powders at 50% RH. b) Corresponding kinetic plots of formaldehyde oxidation. c) Chemoresistive response of flame‐aerosol deposited pure Co_3_O_4_ and Cu/Co_3_O_4_ films at 75°C to 1 ppm formaldehyde at 50% RH as a function of Cu content. The symbol and error bar at 5 wt.% Cu indicate average value and standard deviation of *n* = 3 identically prepared sensors, respectively. d) Scatter plot of sensor response (red circles, left ordinate) and DRIFTS intensity of Cu^+^‐CO vibration (blue triangles, right ordinate, from Figure 3a) over the apparent activation energy (*E*_a_) for formaldehyde oxidation. Spearman's correlation coefficients (*ρ*) are indicated. Note that the dashed line and dash‐dotted line represent the best fit to relate *E*_a_ to sensor response and DRIFTS intensity, respectively.

**Figure** **4**a shows the catalytic oxidation of 1 ppm formaldehyde in air at 50% relative humidity (RH) over pure Co_3_O_4_, CuO and Cu/Co_3_O_4_ powders between 20 – 200°C. For pure CuO and Co_3_O_4_, the formaldehyde conversion starts at 110 and 120°C, while full conversion is only achieved at 200 and 160°C, respectively. Remarkably, the addition of Cu to Co_3_O_4_ significantly reduces these values to lower temperatures. In fact, the best performing 5 wt.% Cu (circles) is already reactive at room temperature (e.g., 37% formaldehyde converted at 40°C with the highest oxidation rate, Figure S8, Supporting Information) and reaches full conversion at only 60°C. Similar, but less effective catalytic performance is obtained for other Cu contents. As a result, 5 wt.% Cu/Co_3_O_4_ features the lowest apparent activation energy (*E*_a_) for formaldehyde of 41 kJ mol^−1^ in comparison to the other compositions (46 – 144 kJ mol^−1^, Figure 4b).

Interestingly, a similar behavior is obtained when these nanoparticles are self‐assembled onto porous films^[^ ^9^ ^]^ and applied then as chemoresistive sensors to detect 1 ppm formaldehyde at 50% RH and 75°C (Figure 4c). Note that 75°C was chosen as the Cu/Co_3_O_4_ compositions yielded high analyte conversions there (Figure 4a). Pure Co_3_O_4_ detects formaldehyde with rather low response (i.e., 0.29). In the case of CuO, no sensor measurements were possible due to too high resistances (i.e., > 1 GΩ) at such low temperatures. Most remarkably, the sensor response increases by up to an order of magnitude compared to pure Co_3_O_4_ when adding Cu, with a maximum at nominal 5 wt.%. This performance is reproducible, as demonstrated with three independently produced sensors at that composition with response variation ≤ 8%. Increasing the Cu content beyond 5 wt.%, however, deteriorates the response.

To elucidate structure‐function relationships further, we show in Figure 4d for each material composition the sensor response to 1 ppm formaldehyde at 50% RH (left ordinate, data from Figure 4c) and the relative amount of reactive Cu^+^ surface sites (i.e., ratio Cu^+^‐CO/CO(g), right ordinate, data from Figure 3a) over their apparent activation energies *E*_a_ for formaldehyde oxidation. Remarkably, the amount of reactive Cu^+^ surface sites (blue triangles) and the sensor response (red circles) are both strongly correlated to *E*_a_ with Spearman's rank correlation coefficients (*ρ*) of 0.89 and 0.96, respectively. As a result, the availability of Cu^+^ surface sites seems a performance descriptor for the catalytic reactivity and sensor response of Cu/Co_3_O_4_ to formaldehyde, enabling its low temperature (e.g., 75°C) detection.

### 2.5 Formaldehyde Detection Limit, Selectivity, and Humidity Effects

**Figure 5:**
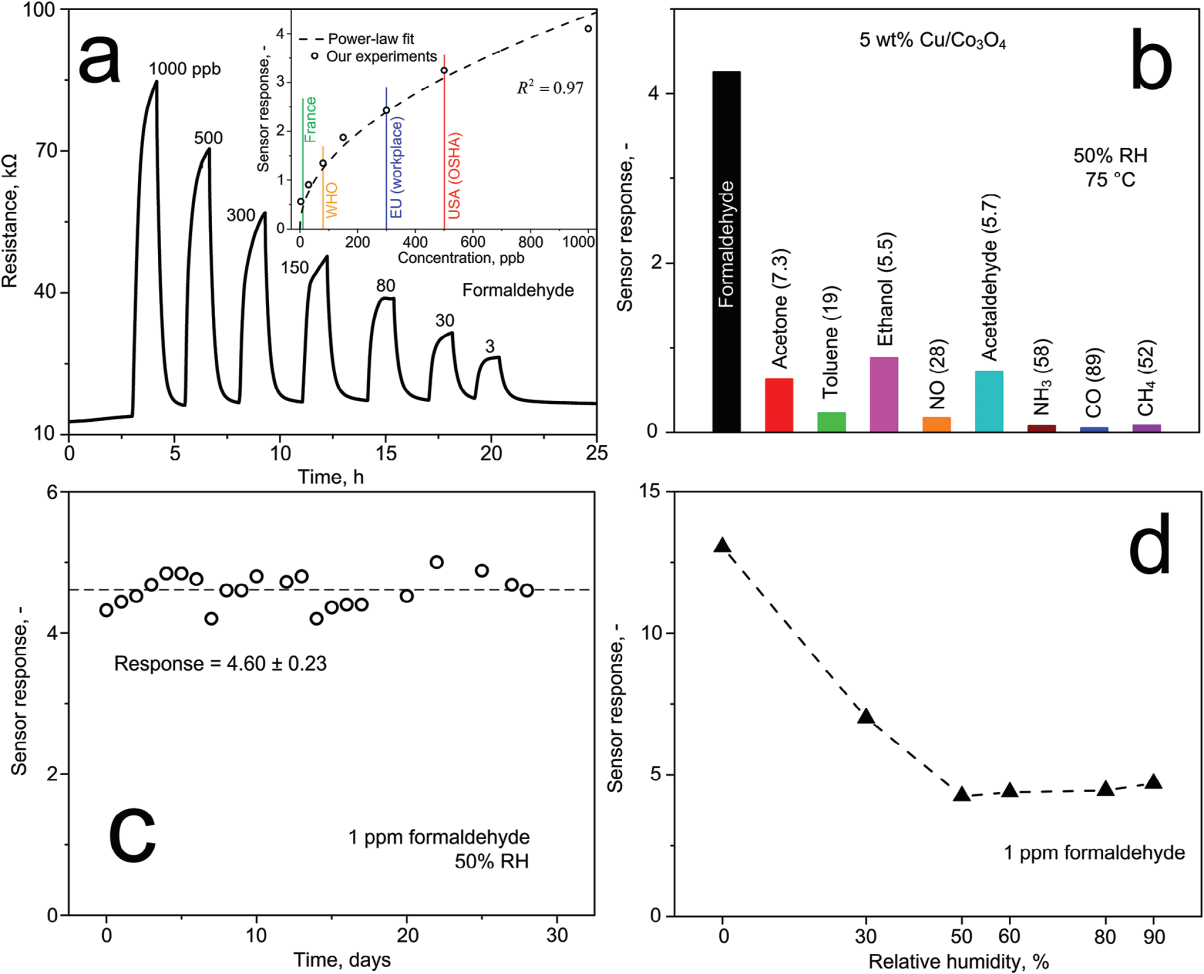
a) Ohmic resistance of a 5 wt.% Cu/Co_3_O_4_ film under exposure to 1000, 500, 300, 150, 80, 30, and 3 ppb of formaldehyde in air at 50% RH and 75°C. Inset shows the corresponding responses (symbols) with indicated exposure limits in the USA,^[^ ^73^ ^]^ EU,^[^ ^74^ ^]^ FR^[^ ^26^ ^]^ and the WHO^[^ ^75^ ^]^ guideline. The dashed line represents a power‐law fit (see Experimental Section). b) Response to 1 ppm formaldehyde and various critical interferents at 50% RH and 75°C, using a 5 wt.% Cu/Co_3_O_4_ film. Catalytic conversions of these analytes are provided in Figure S11 (Supporting Information). c) Response to 1 ppm formaldehyde at 75°C and 50% RH, when measured over 28 days. Indicated are values for average ± standard deviation. d) Response to 1 ppm of formaldehyde under varying RH between 0 – 90%.

Finally, we evaluated the optimal 5 wt.% Cu/Co_3_O_4_ to assess its competitiveness for formaldehyde sensing. A key challenge are the low legal formaldehyde exposure limits that can be as low as 8 ppb in France.^[^ ^26^ ^]^ **Figure** **5**a shows the sensor resistance when exposed to 1000, 500, 300, 150, 80, 30, and 3 ppb of formaldehyde at 75°C and realistic 50% RH. The sensor baseline features a favourably low resistance of ≈15 kΩ, lower than typical flame‐made sensors even at much higher temperatures (e.g., 114 kΩ for Si/SnO_2_ at 400°C^[^ ^65^ ^]^). When exposed to 1000 ppb of formaldehyde, the resistance increases to 84 kΩ, as expected for p‐type chemoresistors when exposed to reducing gases.^[^ ^66^ ^]^ Remarkably, this is clearly distinguished from the other formaldehyde levels and even 3 ppb are accurately quantified with high signal‐to‐noise ratio of 1000.

The sensor response correlates well (coefficient of agreement *R* ^2^ = 0.97) with a power‐law fit (inset, Figure 5a, dashed line). In case of a p‐type semiconductor^[^ ^67^ ^]^ and assuming small grains as well as interaction with O_2_^−^ as reactive oxygen species,^[^ ^68^ ^]^ an exponent *n* = 0.5 was applied. As a result, the sensor response might be related to formaldehyde oxidation through ionosorbed O_2_^−^ species. This can yield a release of surface‐trapped electrons resulting in an increased resistance of our Cu/Co_3_O_4_, in agreement with Figure 5a. Note that the response to small formaldehyde concentrations (e.g., 3 ppb) are a bit higher than the model. Therefore, we performed a control experiment that investigated the effect of blank air and N_2_ samples (contained in the formaldehyde standard, see Methods). Both showed a small interference with the measurement with responses ≤ 0.04 (Figure S9, Supporting Information).

The detection of 3 ppb outperforms previously reported chemoresistive formaldehyde sensors operated below 120°C (Table 1), for instance, Ni‐doped In_2_O_3_/W_2_S^[^ ^69^ ^]^ or 3D ordered macroporous Au/SnO_2_,^[^ ^70^ ^]^ which detected it down to 50 ppb and 10 ppb, respectively, in dry air though. Some sensors detected it down to 10 ppb even in the presence of humidity, like Ti_3_C_2_T_x_ MXene/amino‐functionalized carbon nanotubes^[^ ^71^ ^]^ or MXene/Co_3_O_4_.^[^ ^72^ ^]^ That way, the sensor designed here covers major exposure limits in the USA^[^ ^73^ ^]^ and EU^[^ ^74^ ^]^ as well as guideline values by the WHO^[^ ^75^ ^]^ (inset, Figure 5a). Note that the baseline is always recovered after exposure indicating fully reversible analyte interaction. The response (*t*_res_) and recovery (*t*_rec_) times are between 26 and 51 min at 3 – 1000 ppb of formaldehyde concentrations (Figure S10, Supporting Information), which is sufficiently fast for periodic formaldehyde assessment in critical locations (e.g., freshly renovated houses, hospital pathologies, furniture industry) throughout a day.^[^ ^25^ ^]^ For instance, when switching off ventilation in a whole‐house study in the USA,^[^ ^76^ ^]^ it took about 5.5 h to reach a steady‐state formaldehyde concentration of 56 µg m^−3^ (i.e., 46 ppb) compared to 38 µg m^−3^ (31 ppb) with ventilation. In some applications, faster response and recovery times may be required, that have been achieved by sensors operated at higher temperatures (e.g., Co‐rich ZnCo_2_O_4_^[^ ^77^ ^]^).

Indoor air usually contains >250 kinds of molecules,^[^ ^78^ ^]^ that can interfere with formaldehyde sensing. Figure 5b shows the response of our 5 wt.% Cu/Co_3_O_4_ sensor at 50% RH and 75°C to 1 ppm formaldehyde and various critical analytes covering a wide range of chemical families, including acetone, toluene, ethanol, NO, acetaldehyde, NH_3_, CO, and CH_4_. The sensor features the highest response to formaldehyde, with selectivities between 5.5 for ethanol and 89 for CO. Noteworthy, the response to various analytes follows a similar sequence to catalytic conversion and oxidation kinetics (Figure S11, Supporting Information). In case of CO, higher responses are only observed at elevated temperatures (e.g., 0.33 at 162°C, see Figure S12, Supporting Information), despite its adsorption on Cu^+^ sites even at room temperature (Figure 3a). Probably, an operational temperature of 75°C (Figure 5b) is insufficient to facilitate a reaction, for instance, with ionosorbed oxygen or hydroxyl species^[^ ^79^ ^]^ to yield significant chemoresistive response. In comparison to other formaldehyde sensors (Table 1), our sensor features competitive if not superior formaldehyde‐selectivity that can be further improved, if required, by preceding sorption^[^ ^80^ ^]^ or molecular‐sieving^[^ ^70^, ^81^ ^]^ filters and sensor assembly to arrays, though the latter requires signal processing.^[^ ^65^ ^]^

For air quality, stable performance is required to ensure reliable monitoring over extended periods. Therefore, the 5 wt.% Cu/Co_3_O_4_ was tested over 4 weeks to 1 ppm formaldehyde at 75°C and 50% RH (Figure 5c). The response is quite stable with variation ≤ 5% (standard deviation). This is likely due to tiny changes in the environment (e.g., RH or temperature^[^ ^82^ ^]^) from the gas mixing setup. Importantly, no steady performance fading is observed, which highlights the reversibility of formaldehyde interaction^[^ ^83^ ^]^ and the absence of any surface deactivation of the active CuO_x_ sites.

Humidity is another challenge as it fluctuates in the application. Figure 5d shows the response of 5 wt.% Cu/Co_3_O_4_ to 1 ppm of formaldehyde at 0 – 90% RH and 75°C. In dry air, the sensor shows the highest response (13.1), that decreases by 65% at 50% RH. Most importantly, the response remains largely unchanged between 50 – 90% RH (variation < 4%), which is most relevant for air quality monitoring.^[^ ^84^ ^]^ In comparison to literature, our sensor is more robust to RH (response reduction of 40% in 30 – 90% RH), compared to, for instance, 3DOM Au/SnO_2_ (70%‐reduction in 32 – 91% RH^[^ ^70^ ^]^) and Au@SnO_2_ core‐shell structures (73%‐reduction in 40 – 70% RH^[^ ^85^ ^]^). Residual humidity effects can be compensated by a co‐located RH sensor.^[^ ^86^ ^]^

## 3 Conclusions

We demonstrated the excellent catalytic and sensing properties of CuO_x_ clusters on Co_3_O_4_ nanoparticles at low temperatures. When fabricated by flame‐aerosol technology, finely dispersed clusters were obtained, while some Cu‐ions were incorporated into the Co_3_O_4_ lattice improving thermal stability. Most importantly, detailed material and surface characterization identified Cu^+^ and Cu^2+^ surface species in the CuO_x_ clusters as key reactive sites, with Cu^+^ serving as performance descriptor for catalytic formaldehyde oxidation and its chemoresistive sensing. This was further supported by the reducibility of Cu/Co_3_O_4_ at significantly lower temperatures than pure Co_3_O_4_ or CuO. When dry‐depositing such nanoparticles as porous films, such sensors detected even the lowest formaldehyde concentrations down to 3 ppb already at 75°C, a performance superior to state‐of‐the‐art sensors based on other chemoresistive nanoparticles. Also, good formaldehyde selectivity over other aldehydes, ketones, alcohols, aromatic and inorganic compounds, high humidity robustness and stable performance were observed, rendering such Cu/Co_3_O_4_ promising for air quality control, food safety and health monitoring.

## 4 Experimental Section

### Sensing Nanoparticle Production

Cu/Co_3_O_4_ nanoparticles were prepared by a FSP reactor, as detailed elsewhere.^[^ ^87^ ^]^ Briefly, 5 mL min^−1^ of a liquid precursor were fed through a capillary and dispersed by 5 L min^−1^ O_2_ with a pressure drop across the nozzle of 1.6 bar to form a fine spray. The spray was ignited and sustained by a premixed flamelet of CH_4_ (at 1.25 L min^−1^, Methane 2.5, PanGas, Switzerland) and O_2_ (at 3.25 L min^−1^, Pangas, Switzerland), and shielded with an additional O_2_ sheath flow of 5 L min^−1^. The precursor consisted of cobalt(II) 2‐ethylhexanoate (65 wt.% in mineral spirits, Sigma Aldrich, Switzerland) and Deca Copper 8 (Borchers, Germany), as dictated by the final Cu content. This mixture was dissolved in pure xylene (mixture of isomers, VWR Chemicals, Switzerland) to obtain a total metal (Co + Cu) molarity of 0.2 m. For powders, such made particles were deposited for 15 min onto a water‐cooled glass fiber filter (257 mm diameter, GF6, Hahnemühle Fineart, Germany) at a height above the burner of 57 cm aided by a vacuum pump (Seco SV 1025 C, Busch, Switzerland). The powder was obtained by scraping off particles from the filter with a spatula and subsequent sieving (mesh 300 µm) to remove filter fibers. For sensors, particles were deposited directly by thermophoresis^[^ ^88^ ^]^ for 4 min onto water‐cooled sensor substrates (electrode type #103, Electronic Design Center, Case Western University, USA) at 20 cm height above the burner. The substrates were made of Al_2_O_3_ with interdigitated electrodes on the front and a Pt heater on the back. Powders and sensors were thermally stabilized by a 5‐hour annealing in an oven (CWF 1300, Carbolite Gero, Germany) under ambient air at 500°C. Before first testing, the sensors were heated up to 200°C for 5 min to desorb contaminants from the sensing film.

### Crystallography

XRD patterns of powders were acquired with a Bruker D2 Phaser (USA) operated at 30 kV and 10 mA, at 2θ (Cu K_α_ radiation) between 15° and 75°, with scanning step size of 0.01° and a scanning time of 2.2 seconds per step. Crystal phases were identified by comparison of obtained patterns to the structural parameters of cubic Co_3_O_4_ (PDF 42–1467), monoclinic CuO (PDF 72–0629), orthorhombic Cu_2_CoO_3_ (PDF 76–0442) and cubic Cu_0.92_Co_2.08_O_4_ (PDF 37–0878). All XRD patterns were corrected for displacement with tin telluride (SnTe 99.999%, Sigma Aldrich, Switzerland) as crystalline internal standard.^[^ ^89^ ^]^ Therefore, Cu/Co_3_O_4_ powder was mixed with SnTe in a mortar and its XRD pattern was aligned to the reference peaks of cubic SnTe (PDF 46–1210). Cu incorporation into cubic Co_3_O_4_ was evaluated by peak shift analysis^[^ ^46^ ^]^ of its main reflection at 2*θ* = 36.86° to identify lattice expansion. The refined lattice constant, crystal size (*d*_XRD_) and phase fractions were evaluated with the software Topas 4.2 (Bruker), using the Rietveld fundamental parameter method.^[^ ^90^ ^]^

### Electron Microscopy

Particle images were obtained by TEM and scanning transmission electron microscopy (STEM), performed on a FEI Talos F200X with high brightness gun (XFEG) operated at 200 kV. The particle samples were prepared by dispersing them with ethanol onto perforated carbon foils supported on copper grids. The TEM images and selected area electron diffraction (SAED) patterns were recorded with a CETA CMOS Camera. Energy dispersive X‐ray spectroscopy (EDXS) was conducted in STEM mode with four attached SDD spectrometers (Bruker). High resolution TEM (HR‐TEM) studies were performed using the Grand‐ARM300F (JEOL, Japan) with a cold field emission gun operated at 300 kV. Aberration correction of the image‐forming as well as of the probe‐forming lenses enabled sub‐Angstrom resolution in both TEM and STEM mode.

### Surface Adsorption

The specific surface area (SSA) of powders was determined by nitrogen adsorption (Tristar II Plus, Micromeritics, USA) with a Brunauer‐Emmet‐Teller (BET) 8‐point method. Prior to measurement, samples were degassed for 1.5 h at 150 °C under nitrogen to remove any adsorbate. Surface equivalent diameters (*d*_BET_) were determined with the XRD‐derived phase composition (weight fractions, *w_i_*). The densities (*ρ*) of 6.31 g cm^−3^ and 6.11 g cm^−3^ were used for CuO^[^ ^91^ ^]^ and Co_3_O_4_,^[^ ^92^ ^]^ respectively, that were adjusted for Cu incorporation (as approximation). Thereby, the *ρ* of the composite Cu/Co_3_O_4_ was calculated according to:

(1) $$\rho \left(\frac{g}{{\mathrm{cm}}^{3}}\right)=\frac{1}{\sum_{i\;=\;Cu,Co}\frac{w_{i}}{\rho_{i}\left(\frac{g}{{\mathrm{cm}}^{3}}\right)}}$$

The equivalent diameter *d*_BET_ was calculated through:

(2) $$d_{BET}\left(\mathrm{nm}\right)=\frac{6000}{SSA\left(\frac{m^{2}}{g}\right)\cdot \rho \left(\frac{g}{{\mathrm{cm}}^{3}}\right)}$$

Adsorption of CO^[^ ^51^ ^]^ for CuO_x_ cluster characterization on powders was investigated by DRIFTS using a Vertex 70v spectrometer (Bruker Optics, USA) equipped with a liquid nitrogen‐cooled mercury cadmium telluride (MCT) detector. The powders (4 mg) were mixed with non‐IR active KBr and placed in an in situ DRIFTS cell^[^ ^51^ ^]^ with KBr windows (Harrick Scientific, USA). First, the powder was heated to 100°C for 15 min to desorb any impurities, and then cooled down to 30°C under a 30 mL min^−1^ N_2_ (PanGas, Switzerland) flow, supplied by calibrated mass flow controllers (Bronkhorst, Netherlands). Then, 10 mol% CO in He (PanGas, Switzerland) was supplied at 30 mL min^−1^ and DRIFT absorbance spectra were recorded for 30 min by averaging 150 scans in the range between 700 and 4000 cm^−1^ at 4 cm^−1^ resolution. As discussed in the literature,^[^ ^51^ ^]^ in order to correct DRIFTS of Cu/Co_3_O_4_ for gaseous interference, the spectra are aligned over the broad CO(g) band at 2170 cm^−1^ and normalized to the peak of that band to enable a comparison of relative amount^[^ ^93^ ^]^ of Cu^+^ surface sites.

The redox activity of Cu/Co_3_O_4_ was investigated via H_2_‐temperature‐programmed‐reduction (H_2_‐TPR) with a Belcat‐M instrument (Microtrac MRB, Japan). 20 mg powder was placed in a quartz tube and fixed with wool plugs at both ends to form a packed bed. The bed was first degassed for 30 min at 30°C under Ar (Pangas, Switzerland) at a flow rate of 15 mL min^−1^. Then, the flow was switched to 5 vol% H_2_ in Ar mixture (Pangas, Switzerland) and the powder was heated at a rate of 5 K min^−1^ up to 500°C. The H_2_ consumption by the powder was measured by analyzing the off‐gas of the packed bed with a thermal conductivity detector (TCD). H_2_‐TPR curves were normalized to unity and deconvoluted with Gaussian‐type peaks,^[^ ^64^ ^]^ using the *Multiple‐Peak fit* tool in Origin (OriginLab, USA).

### NAP‐NEXAFS

In situ NAP‐NEXAFS experiments were accomplished at the B07 beamline of Diamond Light Source (UK). The X‐ray was sourced with an energy range from 110 to 2800 eV (soft X‐ray range) and a flux of 1 × 10^10^ photons s^−1^. The endstation consisted of a fixed interface flange which held the entrance cone of the ambient‐pressure electron energy analyzer (SPECS Phoibos NAP‐150, Germany). The samples (around 1 mg) were dispersed in water (around 1 mL) and dropped (around 2 droplets) onto Au‐coated Si (≈1 cm × 1 cm), followed by heating at 70 °C to remove the solvent. NEXAFS spectra at Cu L_3_ edge (924 – 940 eV) were measured in both total electron yield (TEY) mode and Auger electron yield (AEY) mode. The measurements were performed either under UHV or 1 mbar of CO or O_2_. The temperature was monitored by a K‐type thermocouple and regulated by a PID controller.

### Chemoresistive Sensing

The sensors were mounted onto Macor holders and placed in a Teflon‐made chamber, as described elsewhere.^[^ ^94^ ^]^ The sensors were heated by applying a constant voltage to the substrate's Pt heater. The temperature was continuously monitored with a multimeter (2700, Keithley) by using the same Pt heater as the resistance temperature detector (RTD). The sensor chamber was connected to a gas mixing set‐up with inert Teflon tubing, as described elsewhere.^[^ ^46^ ^]^ Briefly, hydrocarbon‐free synthetic air (PanGas, C_n_H_m_ and NO_x_ < 100 ppb) was used as a carrier gas and the analytes from certified gas standards were admixed by calibrated mass flow controllers (Bronkhorst) to obtain the desired gas mixture composition. The gas standards (all Pangas) were: acetone (15.1 ppm), toluene (9.39 ppm), ethanol (14.8 ppm), NH_3_ (10.1 ppm), CO (500 ppm), CH_4_ (10 ppm), methanol (14.3 ppm, all in synthetic air), NO (10 ppm in N_2_), acetaldehyde (17.4 ppm in N_2_), N_2_ (PanGas, 5.0), and formaldehyde (17.3 ppm in N_2_). Humid air was generated by bubbling dry synthetic air through a bubbler filled with de‐ionized water that was admixed to the analyte‐containing gas stream to achieve the desired relative humidity (RH), as checked with a SHT2x sensor (Sensirion AG, Switzerland). The total gas flow rate was 300 mL min^−1^. The ohmic resistance of the sensing film was measured continuously between the interdigitated Pt electrodes with a multimeter (2700, Keithley) to evaluate the chemoresistive sensor response, *S*:

(3) $$S=\frac{R_{g}}{R_{a}}-1$$

where R_g_ and R_a_ are the resistances of the sensing film under gas exposure and in clean air, respectively. Response (*t*_res_) and recovery (*t*_rec_) times are defined as the time needed to achieve 90% of the total resistance change after analyte exposure and removal, respectively.

### Catalytic Conversion

The catalytic conversion was assessed with a custom‐built setup.^[^ ^95^ ^]^ Briefly, 6.4 mg of powder were filled into a glass tube and fixed tightly as a packed bed with quartz wool, as checked visually. The quartz tube was placed in a horizontal oven (Carbolite ESZ 12/450, Germany) and connected to the gas mixing setup, described above for sensing, at a total flow rate of 150 mL min^−1^ and 50% RH. The packed bed was heated between 20 – 200°C, where a heat ramp of 10°C min^−1^ and a dwell time of 20 min at each temperature were applied, before feeding the analyte‐containing mixture. The inlet analyte concentration (*c_in_*) was 1 ppm and the off‐gas (*c_out_*) was analyzed using a proton transfer reaction time‐of‐flight mass spectrometer (Ionicon PTR‐ToF‐MS 1000, Innsbruck, Austria). H_3_O^+^ was used as ion source and the PTR‐ToF‐MS was operated with a drift voltage, temperature, and pressure of 600 V, 60°C, and 2.3 mbar, respectively. The reduced electric field (E/N) in the drift tube was 130 Td.

With deployed catalyst mass and flow conditions, the weight hourly space velocity was kept at 1.4 mL_analyte_ h^−1^ g_cat_^−1^ The analyte concentrations were evaluated at *m/z* values of 31.02 (formaldehyde),^[^ ^96^ ^]^ 33.04 (methanol),^[^ ^97^ ^]^ 45.05 (acetaldehyde),^[^ ^97^ ^]^ 47.05 (ethanol),^[^ ^96^ ^]^ 59.05 (acetone)^[^ ^97^ ^]^ and 93.14 (toluene).^[^ ^97^ ^]^ Before each measurement day, the mass spectrometer was calibrated with 5 points in the range of 0 – 1000 ppb with the aforementioned gas standards for each analyte. The catalytic conversion (*χ*) was defined as:

(4) $$\chi =1-\frac{c_{out}}{c_{in}}$$

The kinetic plots were obtained assuming a pseudo‐first‐order kinetics with respect to the analyte concentration, as commonly observed for such ppm‐level analyte concentrations.^[^ ^98^ ^]^ Therefore, the mass‐based reaction rates were calculated according to:

(5) $$r\left(\frac{\mathrm{mol}}{g_{\mathrm{cat}}\cdot s}\right)=\frac{Q_{tot}\left(\frac{m^{3}}{s}\right)\cdot c_{in}\left(\frac{\mathrm{mol}}{m^{3}}\right)\cdot \ln\left(\frac{1}{1-\chi \;}\right)}{1000\cdot m_{cat}\left(\mathrm{kg}\right)}$$

where *Q*_tot_ is the total inlet volumetric flow, *c*_in_ the inlet analyte concentration and *m*_cat_ the deployed catalyst mass. The activation energy *E_a_* $\left(\frac{\mathrm{kJ}}{\mathrm{mol}}\right)$ was extracted from the temperature‐dependence of the reaction rate, which is an Arrhenius‐type expression according to:

(6) $$r=A\cdot \exp\left(\frac{E_{a}}{RT}\right)$$

Where *A* is the pre‐exponential term (assumed constant), *R* $\left(\frac{\mathrm{kJ}}{\mathrm{mol}\cdot K}\right)$ the universal gas constant and *T* (K) the temperature.

### Statistical Analysis

The mean ± standard deviation (σ) were indicated for experiments that were performed under identical conditions with, at least, three replicates. The Spearman's correlation coefficients (ρ) were determined to assess rank correlation between two variables. Agreement between two variables was determined through the coefficient of determination (*R* ^2^). A power law of the form:

(7) $$S=a\cdot c^{n}$$

was applied to investigate relationships between sensor response (*S*) and formaldehyde concentration (*c*). The exponent *n* was identified from literature, as specified in the Results and Discussions. The parameter *a* was determined by applying the least square method using the Matlab (*The MathWorks, Inc*.) function *lsqcurvefit*.

## Conflict of Interest

The authors declare no conflict of interest.

## Supporting information

Supporting Information

## Data Availability

The data that support the findings of this study are available from the corresponding author upon reasonable request.
