# Supplementary material for: Structure‐Function Relationship of Highly Reactive CuOx Clusters on Co3O4 for Selective Formaldehyde Sensing at Low Temperatures
Source: Adv Sci (Weinh). 2023 Dec 24;11(10):2308224. doi: 10.1002/advs.202308224 (PMC10933674; doi:10.1002/advs.202308224)
Supplement: Supplementary file 1 — Supporting Information [file ADVS-11-2308224-s001.pdf]

ADVANCED SCIENCE
Open Access

Supporting Information

for *Adv. Sci.*, DOI 10.1002/advs.202308224

Structure-Function Relationship of Highly Reactive CuO_x Clusters on Co_3O_4 for Selective Formaldehyde Sensing at Low Temperatures

*Matteo D'Andria, Frank Krumeich, Zhangyi Yao, Feng Ryan Wang and Andreas T. Güntner**

Supporting Information

Structure-Function Relationship of Highly Reactive CuO_x Clusters on Co_3O_4 for Selective Formaldehyde Sensing at Low Temperatures

*Matteo D'Andria, Frank Krumeich, Z. Yao, Feng Ryan Wyan, Andreas T. Güntner**

E-mail: andregue@ethz.ch

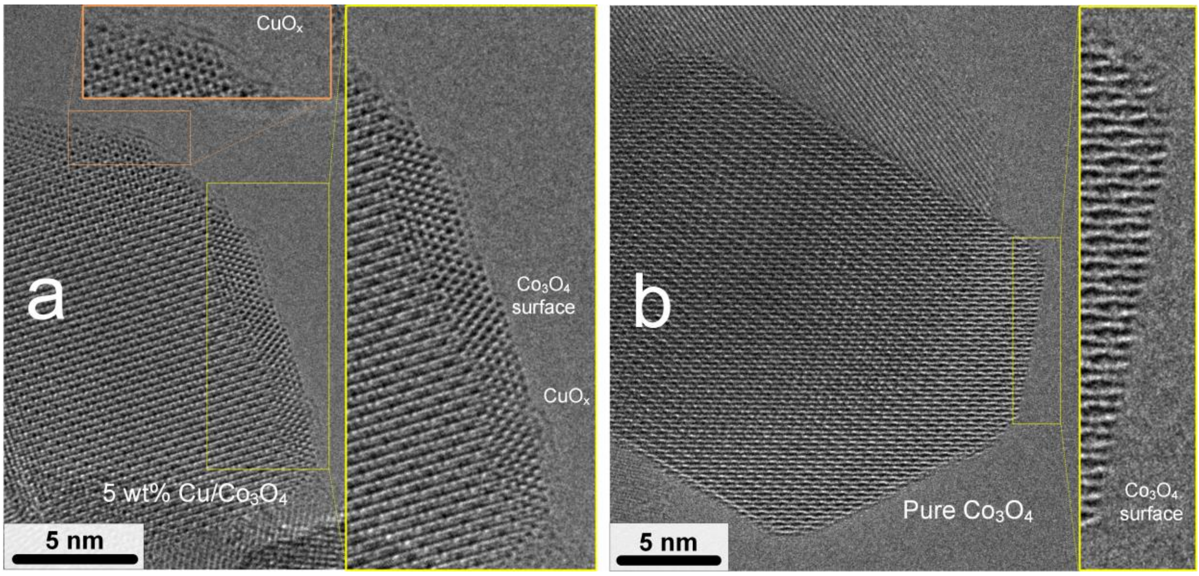

Figure S1: HR-TEM images of (a) 5 wt% Cu/Co_3O_4 and (b) pure Co_3O_4 nanoparticles after annealing at 500 °C for 5 h. CuO_x clusters and cluster-free Co_3O_4 surfaces are indicated and magnified in the insets.

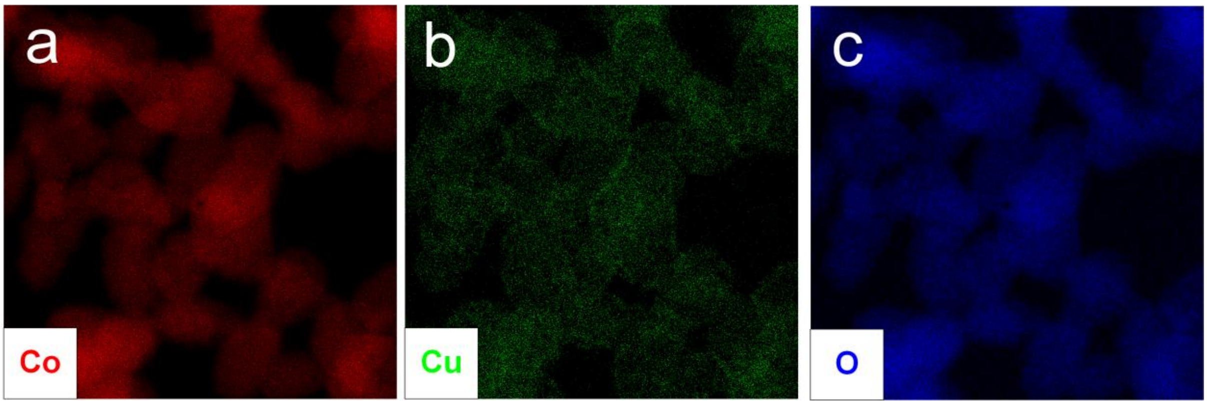

Figure S2: Elemental maps of 5 wt% Cu/Co_3O_4 nanoparticles for (a) Co, (b) Cu and (c) O.

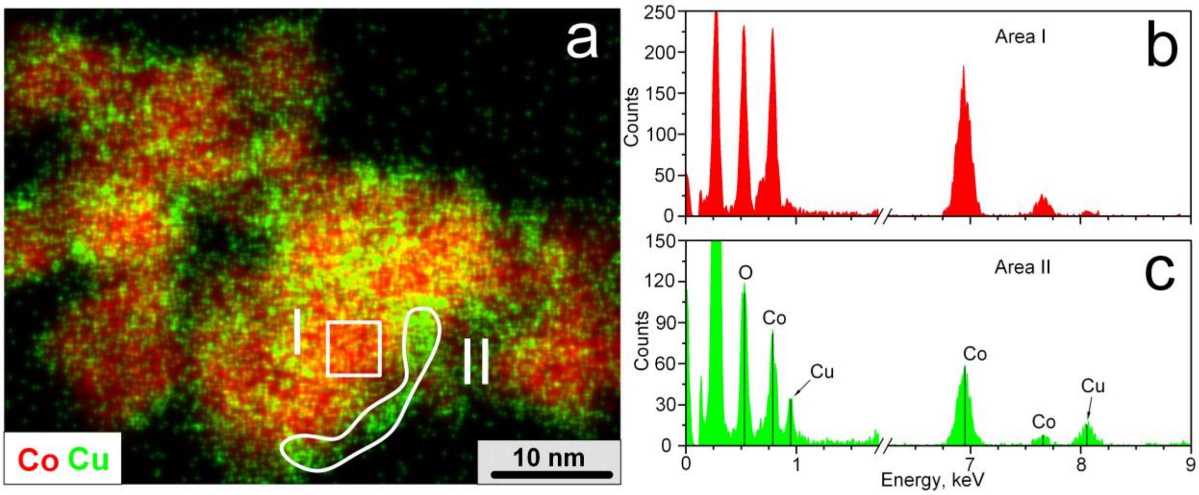

Figure S3: (a) Elemental map of as-prepared 5 wt% Cu/Co_3O_4 with the distribution of Co (red), Cu (green) and O (blue). EDXS spectra of (b) area I and (c) II, as indicated in (a), respectively.

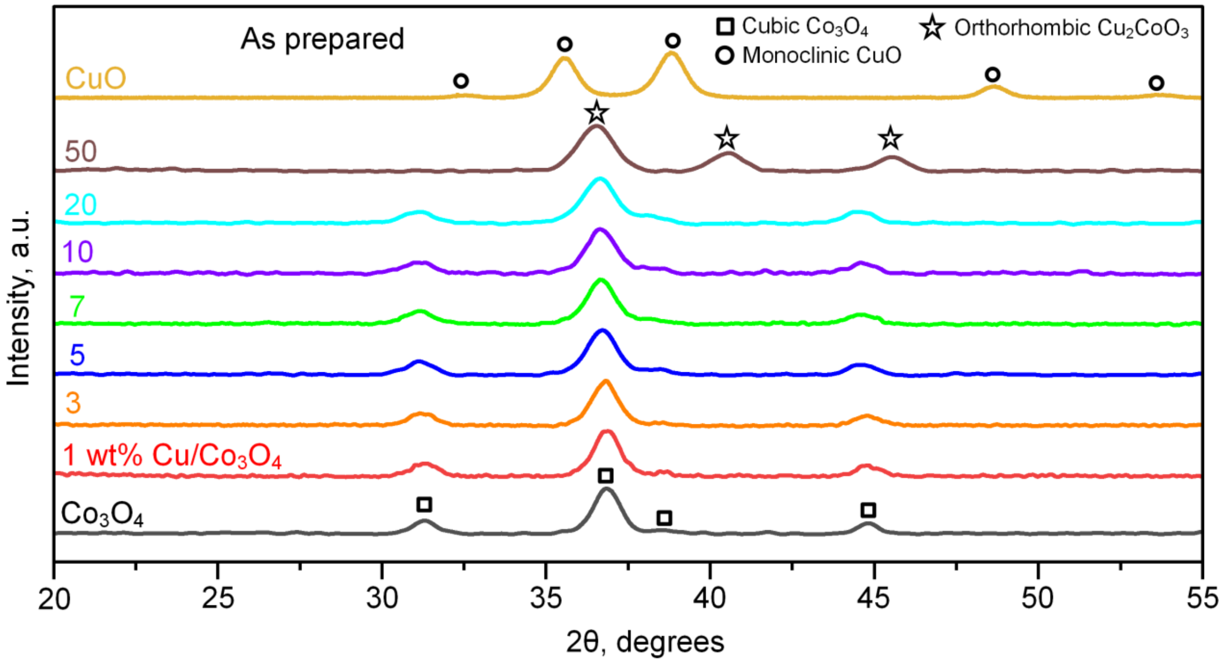

Figure S4: XRD patterns of FSP-made as-prepared pure Co_3O_4, CuO and Cu/Co_3O_4 powders. Indicated are the reference peaks for cubic Co_3O_4 (squares), monoclinic CuO (circles) and orthorhombic Cu_2CoO_3 (stars).

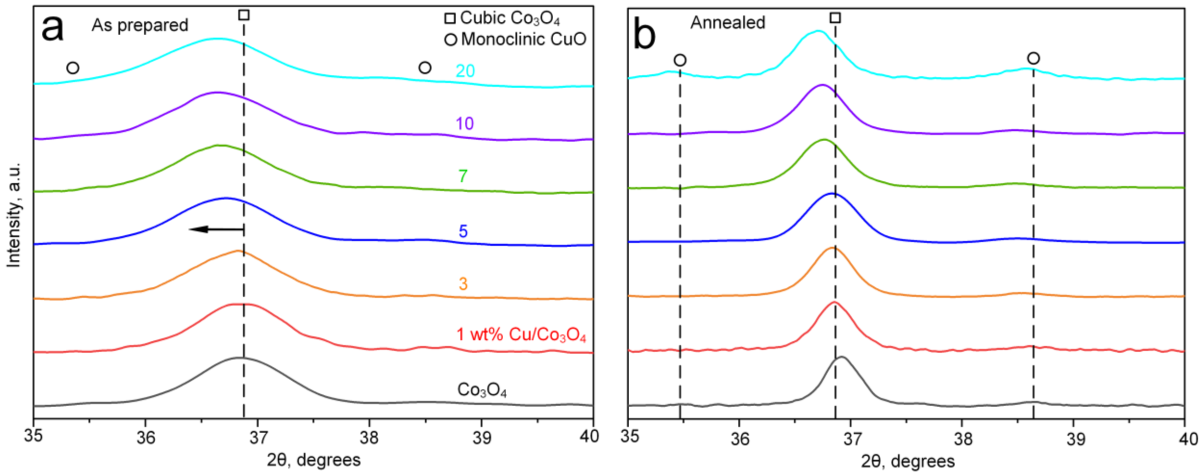

Figure S5: XRD patterns of FSP-made as-prepared (a) and annealed (b) pure Co_3O_4, CuO and Cu/Co_3O_4 powders between 2θ = 35 – 40°. Indicated are the reference peaks of cubic Co_3O_4 (square) and monoclinic CuO (circles).

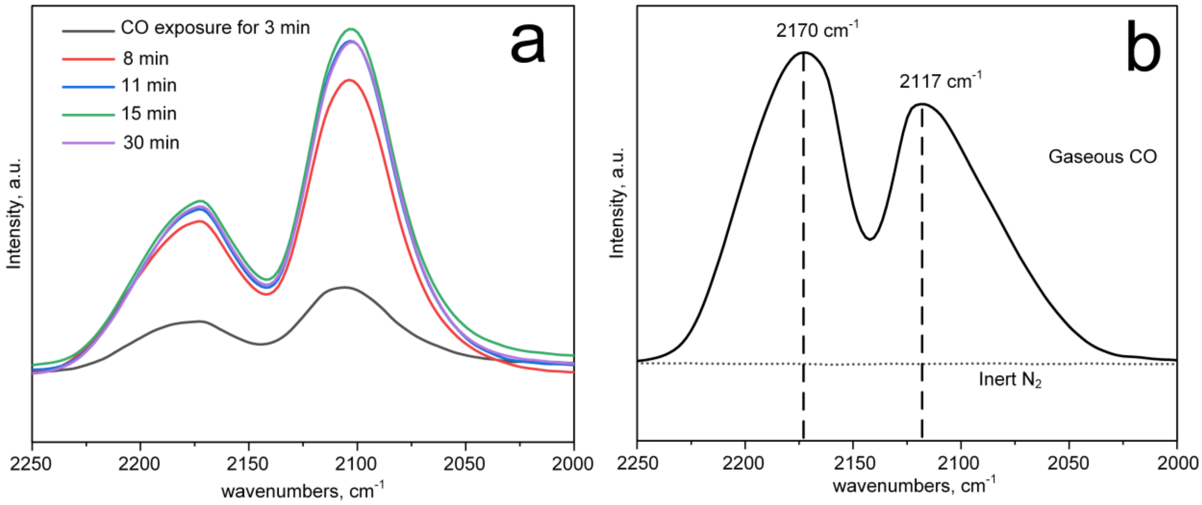

Figure S6: (a) IR spectra of 5 wt% Cu/Co_3O_4 powder after annealing when exposed to CO for 3 - 30 min. (b) IR spectra of inert N_2 (dotted line) and gaseous CO (solid line). Vertical dashed lines indicate the normal vibrational modes of CO(g).

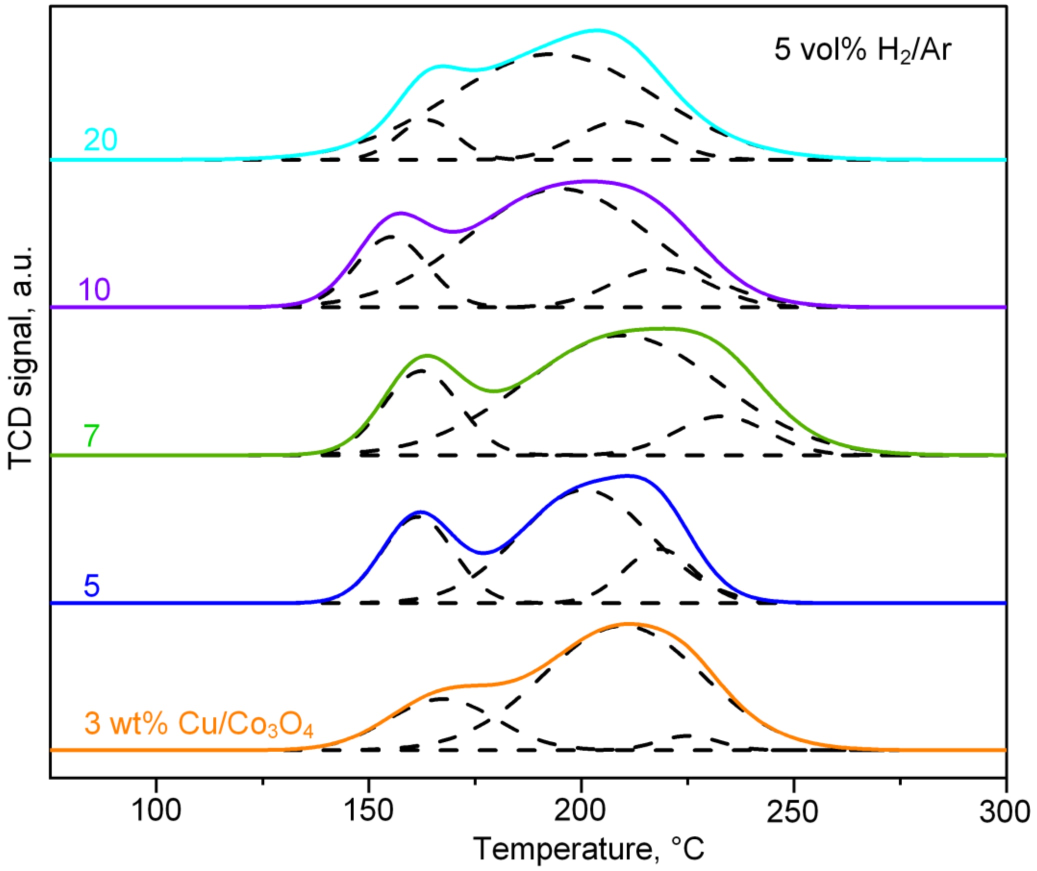

Figure S7: TPR profiles of 3 – 20 wt% Cu/Co_3O_4. Dashed curves indicate the three deconvoluted Gaussian peaks centered around 160, 215 and 230 °C, corresponding to the reduction of Cu^+ and Cu^{2+}, the Cu – [O_x] – Co^{3+} and Cu – [O_x] – Co^{2+} substituted solid solutions, respectively.

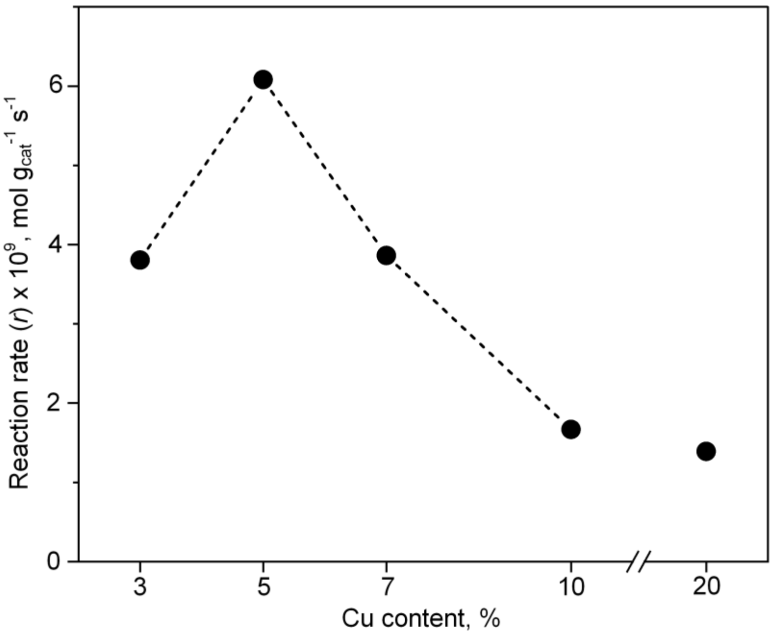

Figure S8: Formaldehyde reaction rate (*r*) at T = 40 °C for varying nominal Cu contents. Note that only *r* for 3 – 20 wt% Cu/Co_3O_4 were measured.

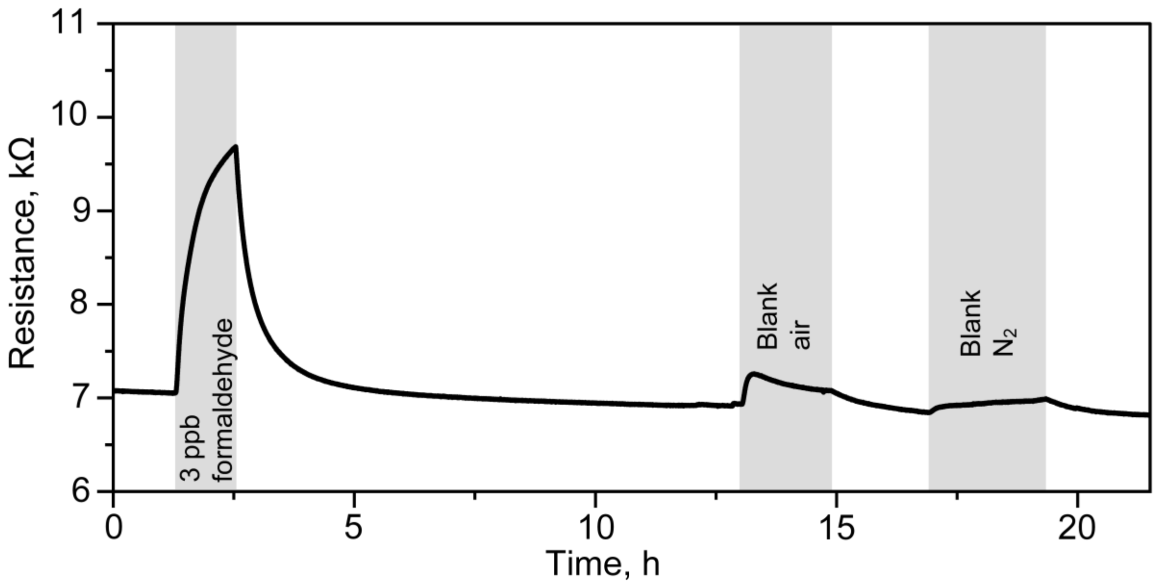

Figure S9: Blank experiment: Ohmic resistance of a 5 wt% Cu/Co_3O_4 film at 75 °C under subsequent exposure to 3 ppb of formaldehyde, synthetic air and N_2 in air (shaded areas) at 50% RH. Measurement was performed by supplying 0.052 mL min^{-1} of these gases subsequently through the same mass flow controller while keeping the overall flow rate at 300 mL min^{-1}. The response to blank air or N_2 was almost an order of magnitude smaller than that to 3 ppb of formaldehyde. Thus, the response to 3 ppb of formaldehyde is predominantly caused by the analyte and other effects are small. These other effects may include, for instance, (1) the use of N_2 as carrier gas of the formaldehyde standard, (2) minor contamination in the synthetic air standard despite hydrocarbon-free grade and (3) possible mass flow controller variations, despite routine calibration.

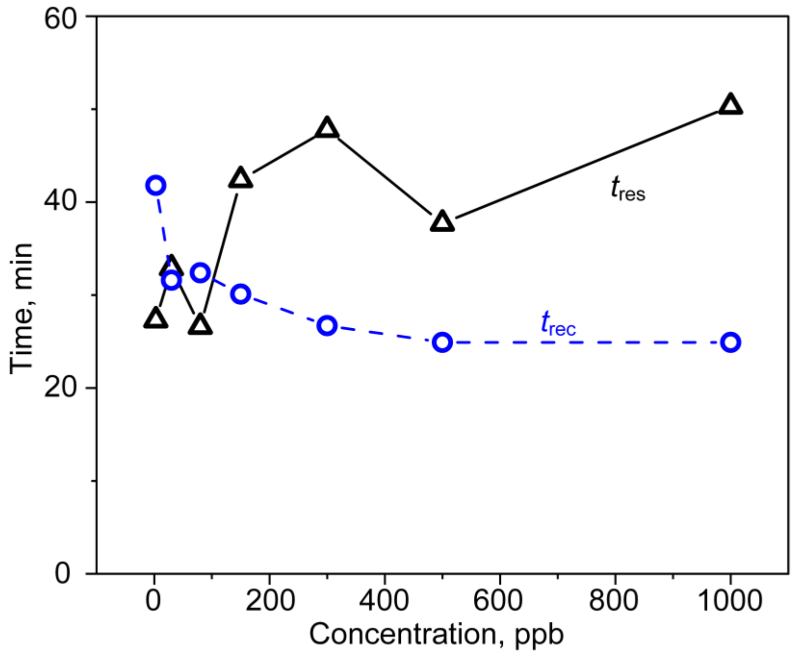

Figure S10: Response (t_{res}) and recovery (t_{rec}) times of a 5 wt% Cu/Co_3O_4 film for 3 – 1000 ppb formaldehyde at 75 °C and 50% RH.

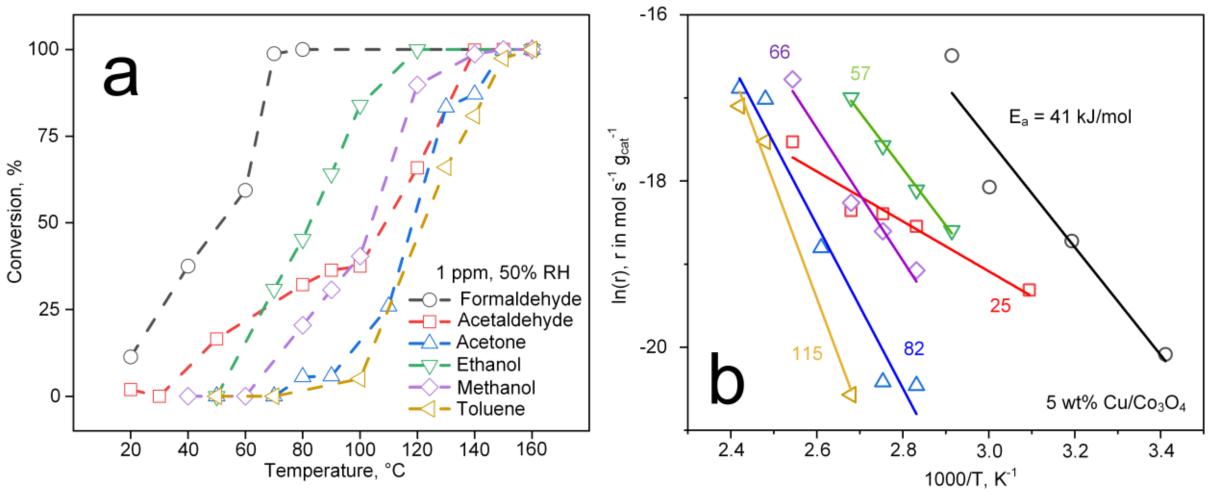

Figure S11: (a) Catalytic oxidation and (b) kinetic plots of 5 wt% Cu/Co_3O_4 powders for the oxidation of 1 ppm formaldehyde and various other molecules at 50% RH.

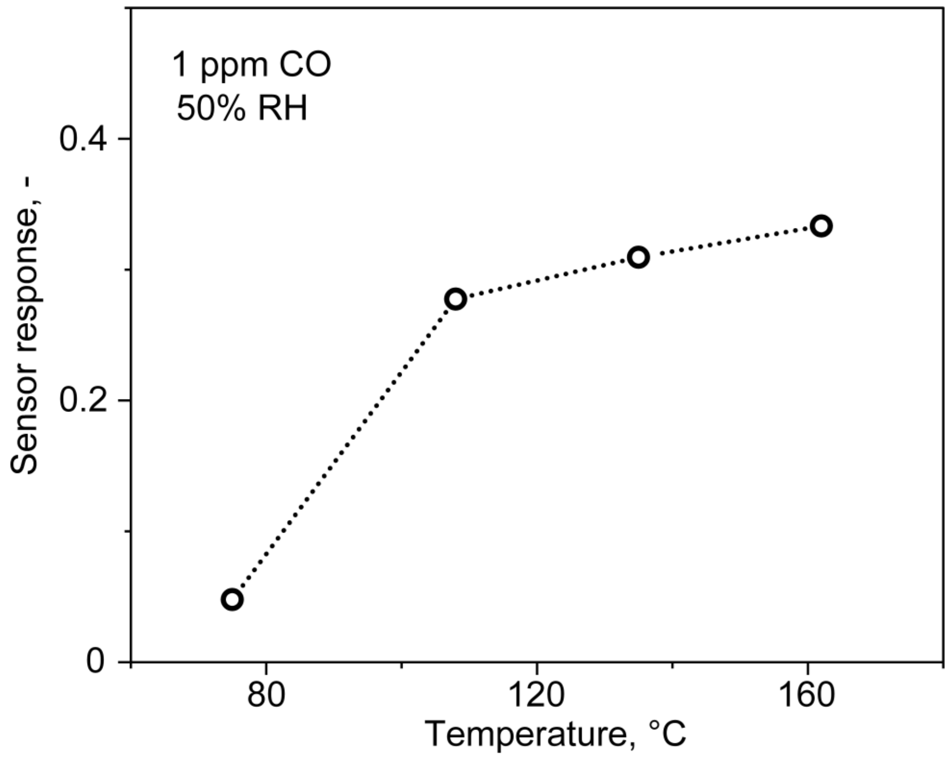

Figure S12: Sensor response of a 5 wt% Cu/Co_3O_4 film at 75 - 162 °C to 1 ppm CO at 50% RH.
